# SFARI Gene 2.0: a community-driven knowledgebase for the autism spectrum disorders (ASDs)

**DOI:** 10.1186/2040-2392-4-36

**Published:** 2013-10-03

**Authors:** Brett S Abrahams, Dan E Arking, Daniel B Campbell, Heather C Mefford, Eric M Morrow, Lauren A Weiss, Idan Menashe, Tim Wadkins, Sharmila Banerjee-Basu, Alan Packer

**Affiliations:** 1 Departments of Genetics and Neuroscience, Albert Einstein College of Medicine, Bronx, NY, USA; 2 McKusick-Nathans Institute of Genetic Medicine, Johns Hopkins University School of Medicine, Baltimore, MD, USA; 3 Zilkha Neurogenetic Institute and Department of Psychiatry and the Behavioral Sciences, Keck School of Medicine, University of Southern California, Los Angeles, CA, USA; 4 Department of Pediatrics, Division of Genetic Medicine, University of Washington, Seattle, WA, USA; 5 Department of Molecular Biology, Cell Biology and Biochemistry; and Department of Psychiatry and Human Behavior, Brown University, Providence, RI, USA; 6 IMHRO/Staglin Assistant Professor, Department of Psychiatry, Institute for Human Genetics, Center for Neurobiology and Psychiatry, UCSF, San Francisco, CA, USA; 7 MindSpec Inc., 8280 Greensboro Drive, Suite 150, McLean, VA, USA; 8 Department of Public Health, Faculty of Health Sciences, Ben-Gurion University of the Negev, Beer-Sheva, Israel; 9 Simons Foundation Autism Research Initiative, New York, NY, USA

## Abstract

New technologies enabling genome-wide interrogation have led to a large and rapidly growing number of autism spectrum disorder (ASD) candidate genes. Although encouraging, the volume and complexity of these data make it challenging for scientists, particularly non-geneticists, to comprehensively evaluate available evidence for individual genes. Described here is the Gene Scoring module within SFARI Gene 2.0 (https://gene.sfari.org/autdb/GS_Home.do), a platform developed to enable systematic community driven assessment of genetic evidence for individual genes with regard to ASD.

## Letter to the Editor

Previous work has crafted curated lists of genes implicated in autism spectrum disorders (ASDs) [1-5], and in some cases developed loosely defined “evidence scores” for entries [6-8]. Such efforts are incomplete, non-systematic and static. Further, this process requires the integration of diverse lines of support that are unequal. What to conclude for a replicated association with *P* <10^-5^? How does this compare to a common variant association not independently replicated, but with rare variants in the same gene? What to make of modest association accompanied by case-control gene expression differences? Given that downstream work involves considerable resources, a portal and framework enabling systematic assessments of all available evidence holds great value.

The Gene Scoring module within SFARI Gene 2.0 was built in an effort to assess the strength of evidence associated with candidate genes and address these concerns. Using a human genetics perspective, we developed a set of criteria to quantify evidence for involvement in the ASDs. These criteria, along with worked examples, are available on the SFARI website (https://gene.sfari.org/autdb/GS_Classification.do). We set out with three guiding principles. First, that relationship to ASD should be based on evaluation of genetic variation in human cohorts. Second, that we start with no assumptions about individual genes. And, finally, that a system to permit active involvement from the scientific community should be at the core of this endeavor. For genes already included in the portal, users with a SFARI login are able to add their own scores alongside curated calls (https://gene.sfari.org/autdb/search); these are viewable by the entire community in the form of “counts”. Registered individuals are also able to propose scores for genes not yet included in the database (https://gene.sfari.org/autdb/user/AddAGene.do), and also suggest modifications to the scoring criteria. Oversight by staff curators and *ad hoc* review by the SFARI Gene Advisory Board together with score histories and a versioning system for scoring criteria will ensure consistency and stability. Although the scoring system we have developed is itself not entirely immune to bias, evidence is examined and applied in a systemic fashion and is sensitive to community feedback.

Using these newly developed criteria, we scored an initial set of 196 genes. Scores and associated annotation were deposited into a newly developed gene-centric web interface. Beyond the gene score itself, a summary of the underlying rationale, links to PubMed and other external databases, functional annotation and a compendium of all identified variants are included. Video tutorials, outlining use of the community annotation interface, have also been developed to facilitate broad uptake (http://www.youtube.com/watch?v=x6PcOXVK0bY). Importantly, all of the underlying data are fully downloadable.

**Figure 1:**
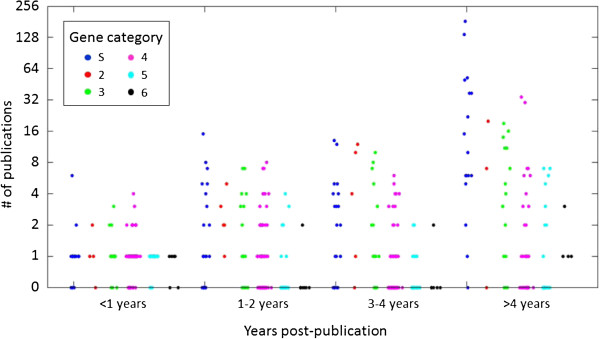
**Weak relationship between strength of ASD-related genetic evidence and the resulting number of scientific publications.** Consistent with a disconnect between attention from the research community and evidence in support of individual genes, the number of publications for individual genes is highly variable and only weakly related to gene category. Publication numbers for each gene were obtained via Pubmed, extracting publications containing the query: "(autism OR autistic) AND ('Gene symbol')" in the title or abstract to obtain a count for each scored gene. To minimize false positives, results were manually curated. Publication number was then plotted as a function of both gene category (colors) and years post publication. ASD, autism spectrum disorder.

Analysis of this initial set of scored genes was revealing (Table 1). A total of 58% of the scored genes, many having been highlighted elsewhere as top candidates, were assigned to the “Minimal Evidence” category. Although scores are not static and will change in the face of new data, these data suggest that there exists only modest support for the majority of autism-candidate genes proposed to date. We then looked at the relationship between category placement and attention from the field, as defined by the number of manually curated publications containing "(autism OR autistic)" in the title or abstract. Enormous variability was observed both within and between categories, along with marked skewing towards specific genes within a given category (Figure 1). Within syndromic genes discovered more than four years ago (n = 17), for example, two genes accounted for almost 50% of the ASD-associated publications. At the other extreme, the eight least attended to from this group collectively accounted for only 8.4% of the publications. This “winner takes most” effect, where two genes attracted almost 50% of the community’s attention, is in direct conflict with a comprehensive understanding of autism genetics. Finally, almost half of the genes with no or relatively modest support (categories 4/5/6) have a greater number of ASD-associated publications than those with more evidence for involvement in disease (categories S/2/3). These data highlight a relatively poor relationship between genetic evidence and attention from the scientific community. It is our hope that SFARI Gene 2.0, together with larger cohorts and greater statistical rigor, will help to highlight those genes with the strongest underlying support.

**Table 1:** More than half of all scored genes were placed within the “Minimal Evidence Category”

**Category**	**Score**	**Number genes**	**Percent genes**	**Average year of discovery**	**Papers observed**	**Papers expected (all years) **^**1**^	**Skewing **^**2**^
Syndromic	S	22	11.2	2003.7	714	160	4.4x
High confidence	1	0	0.0	n/a	0	0	n/a
Strong candidate	2	5	2.6	2007.0	72	37	1.9x
Suggestive	3	17	8.7	2004.4	187	125	1.5x
minimal evidence	4	114	58.2	2007.0	360	834	0.4x
Hypothesized	5	31	15.8	2005.2	82	226	0.4x
Not supported	6	7	3.6	2003.1	18	52	0.3x

^1^ For each category, the percentage of total genes as a function of the total number of papers observed across all categories (n = 1,434).

^2.^Papers observed/papers expected (all years).

Although similar in concept to other efforts, SFARI Gene 2.0 differs in terms of philosophy, methodology and output. To minimize bias, we did not begin with any discussion or review of the literature, but rather the establishment of formalized criteria to quantify available support. Application of these criteria resulted in the identification of genes which despite substantial support had received little or no attention from the field. Also new and unique to SFARI Gene 2.0 is a mechanism that enables researchers to provide reasoned arguments for the introduction of new genes, offer alternate scores that sit alongside existing ones, and provide suggestions for the modification of the scoring criteria. Extensive safeguards - including staff and advisory board oversight - are in place to ensure the quality and utility of the resource.

In summary, key strengths of SFARI Gene 2.0 include explicitly defined scoring criteria, continuous updates and infrastructure to permit community based involvement. We see enormous potential for the resource, a blend of OMIM and Wikipedia, and suggest that it may be useful in helping to ensure the relevance of future computational and functional studies to disease.

## Abbreviations

ASD: Autism spectrum disorder.

## Competing interests

SFARI Gene is licensed by the Simons Foundation from MindSpec. SFARI Gene 2.0 Advisory Board Members have received a yearly honorarium from the Simons Foundation. BSA has also received consulting fees from Integragen, LLC. BSA and DEA also hold patents managed by UCLA and Johns Hopkins University, respectively.

## Authors’ contributions

SBB and AP conceived of and initiated the work. Gene scoring criteria were developed by BSA, DEA, DBC, HCM, EMM, LAW, SBB, and AP. The list of ASD candidate genes complied by IM, TW, and SBB were scoring by BSA, DEA, DBC, HCM, EMM, and LAW, SBB, and AP. Following analyses carried out by all authors, BSA drafted the manuscript, which all authors then edited and revised.
